# Revisiting Platinum-Based Anticancer Drugs to Overcome Gliomas

**DOI:** 10.3390/ijms22105111

**Published:** 2021-05-12

**Authors:** Jaewan Jeon, Sungmin Lee, Hyunwoo Kim, Hyunkoo Kang, HyeSook Youn, Sunmi Jo, BuHyun Youn, Hae Yu Kim

**Affiliations:** 1 Department of Radiation Oncology, Haeundae Paik Hospital, Inje University School of Medicine, Busan 48108, Korea; jjw1066@paik.ac.kr (J.J.); smjo@paik.ac.kr (S.J.); 2 Department of Integrated Biological Science, Pusan National University, Busan 46241, Korea; smlee1048@gmail.com (S.L.); harlemkim@gmail.com (H.K.); kanghk94@gmail.com (H.K.); 3 Department of Integrative Bioscience and Biotechnology, Sejong University, Seoul 05006, Korea; hsyoun@sejong.ac.kr; 4 Department of Biological Sciences, Pusan National University, Busan 46241, Korea; 5 Department of Neurosurgery, Haeundae Paik Hospital, Inje University School of Medicine, Busan 48108, Korea

**Keywords:** platinum-based anticancer drugs, brain tumors, drug resistance, therapeutic efficacy

## Abstract

Although there are many patients with brain tumors worldwide, there are numerous difficulties in overcoming brain tumors. Among brain tumors, glioblastoma, with a 5-year survival rate of 5.1%, is the most malignant. In addition to surgical operations, chemotherapy and radiotherapy are generally performed, but the patients have very limited options. Temozolomide is the most commonly prescribed drug for patients with glioblastoma. However, it is difficult to completely remove the tumor with this drug alone. Therefore, it is necessary to discuss the potential of anticancer drugs, other than temozolomide, against glioblastomas. Since the discovery of cisplatin, platinum-based drugs have become one of the leading chemotherapeutic drugs. Although many studies have reported the efficacy of platinum-based anticancer drugs against various carcinomas, studies on their effectiveness against brain tumors are insufficient. In this review, we elucidated the anticancer effects and advantages of platinum-based drugs used in brain tumors. In addition, the cases and limitations of the clinical application of platinum-based drugs are summarized. As a solution to overcome these obstacles, we emphasized the potential of a novel approach to increase the effectiveness of platinum-based drugs.

## 1. Clinical Treatment for Overcoming Brain Tumors

Gliomas account for approximately 80% of brain tumors. The 5-year relative survival rates of malignant brain tumors were reported to be 34.9%. Among them, the 5-year relative survival rate of glioblastoma was reported to be 5.5% [1]. In 2016, the World Health Organization (WHO) classified central nervous system (CNS) tumors according to molecular biomarkers and clinical phenotypes, in addition to histological classification. In particular, glioblastoma (GBM), which is classified as a grade IV tumor and occurs mostly in adults, has been subdivided to differentiate between different molecular mutations [2,3]. GBM is the most malignant tumor occurring in the CNS, with 54% of gliomas reported to be diagnosed as GBM [4,5,6]. Various treatments have been explored to overcome brain tumors. As a standard therapy, adjuvant chemotherapy and radiotherapy are performed conservatively after surgery [7,8]. Many studies have been conducted to improve the efficacy and reduce the side effects of each therapy, but there are still many obstacles [9,10,11].

Radiotherapy is a standard option for GBM patients along with surgical resection. However, current radiotherapy does not completely eliminate the brain tumors, and the occurrence of radioresistance in surviving brain tumor cells after radiotherapy is regarded as an obstacle to increase therapeutic efficacy [12,13]. In addition, chemotherapy has been used as a combined treatment for brain tumors by targeting cancer cells that cannot be sufficiently removed by surgery [14]. There are several difficulties in increasing the treatment efficacy in brain tumor. One of the reasons is that it is difficult to completely remove tumors in the brain through surgery, and excessive resection can lead to brain dysfunction [15]. Another important reason is the brain tumor microenvironment (TME). Unlike the TME of other tumors consisting of endothelial cells, peripheral cells, fibroblasts, and immune cells, brain TMEs have a unique extracellular matrix and heterogeneous cell types, including microglia, astrocytes, and neurons [16,17]. Since malignant brain tumors have a very strong characteristic of invasion that spreads to the surrounding environment, it is difficult to increase the treatment efficacy and patient survival rate [18]. In particular, glioma stem cells that survive the therapies contribute to self-renewal, proliferation, and differentiation to induce therapeutic resistance and recurrence of brain tumors [19,20,21,22].

To date, various anticancer drugs have been developed and used to treat brain tumor patients. The main key point to brain tumor drug treatment is the contribution of the blood–brain barrier (BBB) to drug delivery. Actually, the clinical application of chemotherapy agents for the treatment of brain tumors is very limited due to the presence of the BBB [23,24]. Temozolomide (TMZ) is the most commonly used anticancer drug against brain tumors because it easily crosses the blood–brain barrier due to its lipophilic nature [25]. TMZ is an imidazotetrazine derivative of the alkylating agent dacarbazine and is orally administered to treat human brain tumors [26]. TMZ was approved by the FDA in 1999 for the treatment of refractory anaplastic astrocytoma in adults and was first applied to patients with GBM in 2005 [27,28]. In the early stages of TMZ application in patients with brain tumors, it increased survival and suppressed tumor growth [29,30]. However, at least 50% of patients receiving TMZ treatment do not respond to this anticancer drug, and several GBM cell lines (U-87, U-251, U-373, T98G) have been shown to contain TMZ-resistant cells [31,32,33,34]. In order to increase the treatment efficacy in malignant brain tumors, overcoming TMZ resistance is important, further studies on xenograft animal models and patient clinical trials are required to investigate TMZ resistance [31,35,36]. Moreover, in order to improve the treatment efficacy, radiotherapy and TMZ have been administered in combination, but the survival rate of GBM patients did not significantly improve with this combined treatment [37,38].

Another option for brain tumor chemotherapy, without definite anticancer drugs, could be platinum-based drugs with established anticancer effects in a variety of carcinomas. The effectiveness of the platinum-based anticancer drug developed by Rosenberg has already been proven in various cancer types such as testicular, ovarian, lung, and head and neck cancer [39,40,41]. A study has been conducted on the clinical effects of three platinum-based anticancer drugs, cisplatin, carboplatin, and oxaliplatin, in brain tumor patients [42,43,44,45]. In recent research, methods to minimize the contribution of the BBB to drug delivery have been explored. These methods include the use of a clinical focused ultrasound device (FUS) and improvement of the delivery system using transferrin-receptor-targeted liposomes [46,47,48]. For about 30 years, there have been no alternative drugs for GBM treatment other than TMZ. Therefore, it is necessary to re-discuss the drugs that have been used in the past as a method to increase the efficacy of brain tumor treatment. In this review, we revisit the benefits of platinum-based anticancer drugs in brain tumor patients and summarize the molecular mechanisms that interfere with the efficacy of chemotherapy.

## 2. Characteristics of Platinum-Based Anticancer Drugs

In 1965, cisplatin was accidentally discovered during Rosenberg’s electric field experiment on the growth of *Escherichia coli* (*E. coli*) bacteria by observing that the platinum conducting plates inhibited cell division. He proved that platinum is important to inhibit bacterial cell division and to stop tumor cell growth [49]. Cisplatin entered clinical trials in 1971, starting with metastatic testicular cancer, ovarian cancer, and bladder cancer [50]. Based on the results of the clinical trials, it was approved by the FDA in 1978. Since the development of cisplatin, thousands of platinum-based analogs have been synthesized and studied to expand the range of treatable tumors [51,52,53].

### 2.1. Anticancer Action Mechanism of Platinum-Based Anticancer Drugs

Cisplatin, carboplatin, and oxaliplatin have been used to treat brain tumors as well as several other forms of cancer, such as ovarian cancer, cervical cancer, and non-small-cell lung cancer [54]. These anticancer drugs react with cancer cells to form identical DNA adducts, but their structures are distinguished and recognized differently [55,56]. Platinum-based anticancer drug–DNA adducts induce various cellular responses such as transcriptional inhibition, cell cycle arrest, DNA repair, and apoptosis [57,58]. Cisplatin forms [Pt (NH_3_)_2_Cl (OH_2_)]^+^ and [Pt (NH_3_)_2_(OH_2_)_2_]^2+^ inside cancer cells, and these forms have the advantage of responding better to DNA, which is the target of anticancer reactions [51,54]. The platinum atom of cisplatin forms a covalent bond at the N7 position of the purine base, forming a crosslink within the 1,2 or 1,3 interstrand. These connections with the DNA of cancer cells form crosslinks with chloride ions or cyclobutane-1,1-dicarboxylate and oxalate ligands in the case of carboplatin and oxaliplatin, respectively [59,60]. Interstrand crosslinks formed by platinum-based anticancer drugs cause structural changes that expose the groove surface and facilitate the binding of various types of proteins [61,62]. Proteins that bind to the modified structure include various repair proteins and transcription factor proteins (HMG domain protein, SSRP1, XPA, hUBF) [63]. In particular, high-mobility group (HMG) box proteins play an important role in platinum-based anticancer reactions [64]. HMG is a group of chromosomal proteins that regulates processes such as transcription, replication, recombination, and DNA repair, and HMGB1 is the most abundantly present protein and is involved in several DNA-related signaling pathways (NF-κB, MAPKs) [65,66,67,68]. HMGB1 recognizes DNA damaged by platinum-based anticancer drugs and regulates the efficiency of nucleotide excision repair. It also directly interacts with the tumor suppressor protein p53 and enhances the p53–DNA binding activity [51,69]. In addition, HMGB1 plays an important role in repairing mismatched DNA by interacting with MutSɑ, a DNA mismatch repair protein [70], and is also involved in the DNA damage signaling pathway mitogen-activated protein kinase (MAPK)-nuclear factor kappa B (NF-κB) [71,72]. Collectively, platinum-based anticancer drugs inhibit the transcription of tumor cells and induce cell cycle arrest through DNA adducts generated by the reaction. DNA adducts by platinum-based drugs are primarily involved in the DNA repair process and induce apoptosis.

### 2.2. Efficacy and Advantages of Platinum-Based Anticancer Drugs

Metal-based drugs have continued to play an important role in overcoming cancer. Platinum-based anticancer drugs attack a single target, cellular DNA, and their direct coordination of nucleobases to nucleophilic nitrogen plays an important role in the induction of tumor cell apoptosis [73]. Many platinum complexes have been designed to optimize platinum–DNA interactions, and increasing their affinity for DNA reduces the exposure of platinum to other cellular nucleophiles [74,75]. This effect can lead to both the reduction of side effects and overcoming tolerance based on the increased glutathione concentration, which can enhance treatment efficiency [76,77]. Another reason for the effectiveness of platinum-based drugs is the enhanced permeability and retention effect (EPR effect) [77]. Tumors are often hyperpermeable to macromolecules as a result of damaged vascular structures, and an accumulation of macromolecules can occur due to lack of efficient lymphatic drainage [78]. These EPR effects have been used in the development of platinum-based anticancer therapies. For example, the poor water solubility and low lipophilicity of various liposomal formulations of platinum-based drugs overcome the difficulty of efficiently encapsulating drugs in liposomes [79,80,81,82]. In addition, since drugs are composed of vector ligands, when administered, they are rapidly distributed throughout the body, interacting with all cancer cells [77,83]. Taken together, the advantage of platinum-based drugs is that the platinum complex structure was designed to optimize DNA interaction and the EPR effect was used in drug development.

### 2.3. Limitations of Platinum-Based Anticancer Drugs

Platinum-based anticancer drugs, which exhibit excellent drug responses, also have side effects and drug resistance, similar to many other anticancer drugs. Approximately 40 side effects of platinum-based anticancer drugs have been reported, and as a side effect of drug toxicity, these include gastrointestinal toxicity, including in the mucous membranes of the mouth, throat, stomach, and intestines [84,85,86]. Cisplatin and carboplatin are known to exhibit nephrotoxicity (kidney damage) and hepatotoxicity (liver damage) [87,88]. In particular, nephrotoxicity is an obstacle to continue treatment when cisplatin is combined with radiotherapy [89]. In addition, cardiotoxicity, ototoxicity, and hematological toxicity have been reported in clinical trials [90,91,92]. The three platinum drugs that are widely used against brain tumors have remarkable effects, but their use is restricted by the number and severity of side effects, and there are limitations in increasing treatment efficiency [84]. Furthermore, the factor that interrupts the increase in chemotherapy efficiency is drug resistance, and the causes of platinum-based drug resistance are summarized in Section 4.

## 3. Application of Platinum-Based Anticancer Drugs in Clinical Treatment

### 3.1. Clinical Trials of Cisplatin in Brain Tumor Treatment

Cisplatin, a first-generation platinum-based drug, has been used in chemotherapy for brain tumor patients along with other cancer treatment. Sheleg et al. performed local chemotherapy and radiotherapy using cisplatin in 38 GBM patients and analyzed the treatment results. The patients were divided into two groups: the control group receiving only partial tumor resection, and the experimental group receiving cisplatin and irradiation after subtotal tumor removal. A total of 20 polymer plates (1.5 × 1.5 cm) incorporated with cis-diaminedichloroplatinum (TEVA Pharmaceutical Industries Ltd, Petach Tikva, Israel) (total cisplatin dose 45 mg) were implanted into the tumor bed [93]. In addition, radiation therapy was administered with a daily dose of 2 Gy in 30 fractions, totaling to a radiation dose of 60 Gy to the cranial area. According to the presented brain CT results, cisplatin biodegradation occurred 5 weeks after implantation into the removed tumor layer wall, and it was confirmed that tumor growth was suppressed compared to the control group. In addition, follow-up of patient survival showed that topical chemotherapy of cisplatin combined with radiation therapy significantly increased the median survival (days 211.0 to 427.5, *p* = 0.00001) [93]. These clinical results provide important evidence for cisplatin’s radiation sensitization effect in postoperative radiotherapy [94,95]. In 2019, Hall et al. showed clinical effects when pegargiminase (ADI-PEG20) was combined with cisplatin in recurrent high-grade gliomas. Ten patients with a median enrollment age of 51 years (43–63 years) were included, and ADI-PEG 36 mg/m^2^ and cisplatin 75 mg/m^2^ were administered intravenously once every 3 weeks for up to 6 cycles. Decreased gliomas was seen on the presented MRI scan, and the overall survival period was increased to 6.3 months, with a median value of 5.6 months [96].

### 3.2. Clinical Trials of Carboplatin in Brain Tumor Treatment

Carboplatin, a second-generation platinum-based anticancer drug, was used in the first clinical trial in pediatric brain tumor patients in 1990 [97]. At that time, cisplatin also had reported effects in several pediatric brain tumors, but studies showed various side effects and cytotoxicity that lead to the interruption of treatments [98,99]. Gaynon et al. studied 95 patients with brain tumors aged 1–20 years (median 7 years). The children with brain tumors were administered carboplatin 560 mg/m^2^ every 4 weeks. As a result of the clinical trial, the complete response (CR) and partial response (PR) were evaluated according to the extent of lesion disappearance using CT imaging and MRI, and approximately 40 brain tumor patients showed reduction in tumor volume [97]. There have also been studies showing that carboplatin increases the clinical treatment efficacy when combined with radiation therapy. Allen et al. administered carboplatin with radiotherapy in 34 glioma patients [100]. Radiotherapy was performed twice a week on schedule, and a total of 72 Gy was irradiated by the hyperfraction method, with a dose of 100 cGy twice a day, and several steps were devised to determine the maximum tolerated dose of intravenous carboplatin, with total cumulative dose of 1540 mg/m^2^ over 7 weeks. The results of this combination treatment were evaluated in 29 of 34 patients by comparing MRI scans before and after treatment, and a good objective response was observed in 15 patients (52%). Additionally, the median progression-free survival of the participating study patients was 8 months, and the median overall survival was 12 months [100]. Based on the results of clinical studies, carboplatin acts as a radiation sensitizer, and it can increase the treatment efficacy in brain tumors [101].

### 3.3. Clinical Trials of Oxaliplatin in Brain Tumor Treatment

Oxaliplatin, developed by Kidani, has increased patient survival more than irinotecan and capecitabine in many clinical studies and has shown a superior anticancer response [102,103]. Oxaliplatin has been shown to be active in cisplatin-resistant cells and is a potentially attractive substance for use in childhood malignancies because of its proven platinum activity in many pediatric tumors [104,105]. Fouladi et al. confirmed the effectiveness of oxaliplatin in recurrent and refractory pediatric brain tumors [106]. The clinical trial was an open-label phase 2 study of oxaliplatin, which was performed in children with recurrent or refractory medullary tumors and upper primitive neuroectodermal tumors. A total of 43 pediatric brain tumor patients with a mean age of 8.5 years (6–18.9 years) were enrolled, with 130 mg/m^2^ of oxaliplatin intravenously injected every 3 weeks over 2 h. To evaluate the clinical effect of oxaliplatin, tumor size was measured in two vertical directions using MRI. The evaluation criteria for brain tumor size change were CR, PR (defined as a >50% decrease in the maximum perpendicular diameters of the tumor). As a result of the evaluation, there was no CR. However, 15 patients showed a PR (13.3%, CI_0.95_ 2.4%–38.4%) reaction. Compared to cisplatin and carboplatin, oxaliplatin is well tolerated in pediatric brain tumor patients, but the anticancer drug response is limited in children with recurrent CNS tumors [97,99,106]. Maindrault-Goebel et al. confirmed the clinical efficacy of oxaliplatin in patients with pediatric malignancies with an average age of 12 years (2–16 years). Tumor types included hepatoblastoma and sarcoma, and CNS tumors included medulloblastoma, diffuse cranial glioma, and high-grade glioma. In this clinical trial, oxaliplatin was used together with 5-fluorouracil (5-FU) and leucovorin (LV), which have been proven to be effective in colorectal cancer [107,108]. Oxaliplatin was administered in two groups (85 and 100 mg/m^2^), and 5-FU was administered from 400 mg/m^2^ to 2400 mg/m^2^ for 2 weeks. The LV dose was fixed and administered at 400 mg/m^2^ in all cohorts. Based on radiological studies (CT, MRI, bone scan), tumor size measurements and blood tests were performed to confirm the clinical effectiveness. Two patients with CNS tumors at the second dose level showed stable disease during the treatment period, and one patient with diffuse pontine glioma showed an 85% tumor necrosis response. Based on this study, FOLFOX therapy, in combination with oxaliplatin and LV, 5-FU, appears to be an effective treatment for refractory solid tumors, including CNS tumors [109]. The contents and results of clinical trials of platinum-based anticancer drugs applied to brain tumor treatment are summarized (Table 1).

## 4. Resistance Mechanism of Platinum-Based Anticancer Drugs in Brain Tumors

### 4.1. Molecular Mechanisms for Cisplatin Resistance in Brain Tumors

Cisplatin has been proven to have excellent clinical efficacy. However, resistance to chemotherapy occurs inside cancer cells, reducing treatment efficiency, and platinum-based chemotherapy is no exception [110]. Overcoming drug resistance by revealing the molecular mechanisms that mediate cisplatin resistance will have important clinical value in improving the treatment outcome of brain tumors [111,112]. Many research have been conducted on the mechanism of cisplatin resistance acquisition in brain tumor cells. Jiang et al. found that DANCR, a long-coding RNA, was negatively correlated with cisplatin sensitivity in glioma cells. DANCR has been reported to play an oncogenic role in various cancer cells by increasing stemness features and promoting tumor progression [113,114]. In addition, recent studies have reported that inhibition of DANCR affects cancer cell progression, invasion, and migration in many carcinomas [115,116]. However, the role and underlying molecular mechanisms of DANCR in cisplatin resistance in glioma cells remain unclear. It was found that DANCR attenuates cisplatin-induced apoptosis in human glioma cells [112,117]. DANCR also upregulates AXL by competitively binding with miR-33a-5p, miR-1-3p, miR-206, and miR-613 [118]. By upregulating AXL, DANCR activated the PI3K/Akt/NF-κB signaling pathway in glioma cells, and inhibition of this signaling pathway reversed the effect of DANCR on cisplatin resistance [119,120]. Based on these results, DANCR promotes cisplatin resistance by activating the AXL/PI3K/Akt/NF-κB signaling pathway in gliomas [112].

Another reason for cisplatin resistance was found to be related to glutathione S-transferase pi 1(GSTP1). GSTP1 is phosphorylated and functionally activated by the serine/threonine kinases of the protein kinase C (PKC) class [121,122]. It has been shown that the activation of phosphorylated GSTP1, which catalyzes the conjugation of cisplatin and glutathione, correlated significantly with increased glutathionyl-platinum formation, decreased DNA strand crosslink formation, and increased cisplatin resistance [123,124]. Additionally, the combined inhibition of PKCa and GSTP1 had a synergistic effect, indicating a higher cisplatin sensitivity. Apoptosis induced by cisplatin activated the translocation of Bax to mitochondria and cytochrome c and caspase-3/7 signaling, and the levels of Bax and cytochrome c by GSTP1 were significantly different [125]. These findings support that PKCa-dependent phosphorylated GSTP1 is associated with cisplatin resistance mediated by cisplatin metabolism [126,127].

The findings presented by Lai et al. show that cisplatin drug resistance in glioma is associated with microRNAs (miRNAs). Differential expression of miRNAs exists in various cancers, including glioma, and their counterparts [128,129,130]. The miR-873 presented in this study was differentially expressed in cisplatin-resistant glioma cells than in wild-type glioma cells. The expression of miR-873 decreased in a time-dependent manner following cisplatin treatment. In addition, analysis of luciferase assay confirmed that Bcl-2 was a direct target of miR-873 and showed that miR-873 negatively correlated with Bcl-2 reduction in cisplatin-resistant glioma cells [131]. Rocha et al. have shown that cell survival due to treatment with cisplatin is independent of the p53 status of glioma cells. On the other hand, it was shown that the concentration of glutathione in glioma cells affects cisplatin resistance and plays an important role in activating the caspase-3/7 apoptosis pathway [132]. Moreover, studies by Park et al. have shown that cisplatin resistance in glioblastoma cells is related to the mitogen-activated protein kinase phosphatases (MKPs) mediated by the JNK signaling pathway [133]. MKPs dephosphorylate the tyrosine and serine/threonine residues of MAPKs, and MAPK activity is regulated by MKPs [134,135]. MKPs are divided into three groups, one of which is MKP-1, which is localized to the nucleus. MKP-1 has high specificity for MAPKs, including p38MAPK and JNK [136,137]. According to research, the expression of MKP-1 was high in glioblastoma cell lines treated with cisplatin. These results indicate that MKP-1 is highly correlated with tumor progression and contributes to drug resistance in glioblastoma [138]. In addition, it was confirmed that overexpressed MKP-1 increases resistance to platinum-based anticancer drugs through the inhibition of JNK phosphorylation [133].

### 4.2. Molecular Mechanisms for Carboplatin Resistance in Brain Tumors

Carboplatin, an analog of cisplatin, binds to DNA and inhibits replication and transcription, causing apoptosis, and is known to have fewer side effects than cisplatin [139,140]. Several studies have been conducted on the molecular mechanisms to increase the efficiency of chemotherapy by lowering the resistance to carboplatin. Laplante and Sabatini found that the mammalian target of rapamycin (mTOR) signaling pathway is responsible for carboplatin resistance in pediatric low-grade glioma. The mTOR pathway regulates important cellular functions, such as cell cycle progression and metabolism [141]. In the study, when mTOR signaling was suppressed, the MAPK pathway was activated and the sensitivity to carboplatin increased. Inhibition of mTOR signaling was confirmed to increase the efficacy of carboplatin in glioma cells by reducing the glutathione pool [142]. As a basis for this, tumorigenesis in glioblastoma multiforme is associated with abnormal PI3K/AKT/mTOR signaling, and inhibition of mTOR signaling induces the activity of MAPK(pERK1/2) and MEK1/2 [143].

According to Seo et al., c-FLIP and Mcl-1 are proteins involved in carboplatin resistance in head and neck squamous cell carcinoma cells and glioma cells. When carboplatin was administered, c-FLIP and Mcl-1 were downregulated, and caspase-mediated cell death was inhibited. In addition, it was suggested that c-FLIP and Mcl-1 were downregulated when treated with carboplatin in glioma cells was because of the increased activity of proteasome subunit alpha 5 (PSMA5) [144]. Furthermore, the nuclear erythroid 2-related factor (Nrf2)/antioxidant response element (ARE) signaling pathway is involved in the regulation of PSMA5 expression [145,146]. The Nrf2/ARE signaling pathway is responsible for the expression of many genes involved in cellular antioxidant and anti-inflammatory defense [147]. Thus, the molecular mechanism of carboplatin resistance occurs through the regulation of Mcl-1 and c-FLIP by the elevation of Nrf2-dependent PSMA5 expression in glioma cells. Recently, there has been a study related to Fanconi anemia group D2 protein (FANCD2) as a modulating factor of carboplatin sensitivity in pediatric high-grade glioma (pHGG) [148]. pHGG is characterized by epigenetic alterations and defects in DNA damage repair genes and causes the occurrence of malignant brain tumors due to a syndrome called Fanconi anemia, a genetic disorder that results from disruption of the Fanconi DNA repair mechanism [149,150]. FANCD2, one of the key components of the Fanconi DNA repair mechanism, forms a complex with FANCI to induce an intracellular response to DNA damage [151]. In addition, this protein is ubiquitinated in response to DNA damage and acts as a regulator of BRCA1 and BRCA2, which are involved in DNA repair [152]. Dennis et al. confirmed that FANCD2 is overexpressed in HGG and that FANCD2 is depleted by its interaction with carboplatin, which induces cytotoxicity. In addition, carboplatin induced sensitivity to FANCD2-dependent DNA crosslinking in glioma cells. Thus, these findings show that FANCD2 regulation acts as a strategy to overcome chemical resistance in pHGG [148,153].

### 4.3. Molecular Mechanisms for Oxaliplatin Resistance in Brain Tumors

Oxaliplatin, which contains platinum along with oxalate and diaminocyclohexane, has been shown to induce a variety of antitumor effects as well as DNA crosslinking, a traditional platinum-based drug damage mechanism [154]. This effect is called the multifaceted alternative effect. Oxaliplatin induces several anti-tumor effects, including modulation of cytokines, transcription factors, and tumor immune suppression mechanisms [155,156,157,158,159]. Oxaliplatin has been shown to induce a better alternative effect than cisplatin and carboplatin, and signal transducer and activator of transcription 3 (STAT3), a key transcription factor in GBM pathology, is being studied as the main signaling mechanism [160,161,162]. A 2018 study by Nathan B. Roberts suggested that oxaliplatin has powerful anticancer effects on malignant glioma cells by reprogramming the microenvironment, proposing that the main regulator of this process is STAT3 [162]. STAT3 was overexpressed in 90% of GBM patients [163], and the effect of STAT3 inhibition on the sensitivity of oxaliplatin treatment was shown in a previous GBM study [162]. The STAT3 signaling pathway is involved in a variety of cellular activities in glioma, including angiogenesis, invasion, chemotherapy resistance, and immune suppression [164,165]. In addition, STAT3 regulates and selects macrophages, which are primary glioma-infiltrating immune cells, and STAT3 is attracting attention as a major regulator of GBM anticancer effects [163,166,167].

The most recent study on oxaliplatin resistance was carried out using head and neck carcinoma cells, CNE1 and CNE2. Chi et al. suggested that Taxol-resistant gene 1 (Txr1) is a causative factor for oxaliplatin resistance [168]. They found that the mRNA and protein expression of Txr1 was increased in oxaliplatin-resistant CNE1 and CNE2 cells compared to that in the parental cells, and the cause of Txr1-mediated resistance was associated with increased autophagy [169]. Autophagy is an important process that constitutes intracellular regulatory mechanisms that maintain cellular homeostasis [170,171]. Moreover, Txr1 induces resistance to oxaliplatin by promoting autophagy through modulation of MEK/ERK signals. In previous studies, abnormal MEK/ERK signaling has been reported in several types of carcinoma [172], and cell proliferation, metastasis, and drug resistance have been reported to be associated with the MEK/ERK signaling pathway [173,174]. Thus, resistance to oxaliplatin can be overcome through autophagy deficiency induced by Txr1.

In summary, DANCR and miR-873 and Mkp-1, molecular factors inducing cisplatin resistance, are primarily involved in the PI3K/Akt/NF-κB and Bcl-2 signaling pathway. Additionally, c-FLIP and FANCD2, molecular factors inducing carboplatin resistance, are involved in the MAPK and Nrf2/ARE signaling pathway and DNA repair processes. Finally, the main cause of oxaliplatin resistance is activation of STAT3 signaling pathway.

## 5. A Strategy for Improving the Efficacy of Platinum-Based Anticancer Drugs in Brain Tumors

Drug resistance is a major complication of chemotherapy and acts as an obstacle to improving platinum-based anticancer therapeutic efficacy in most carcinomas and brain tumors [175,176]. Drug resistance must be overcome in order to increase the therapeutic effect of the anticancer drugs developed till date. One of the methods of overcoming resistance to anticancer drugs is to use inhibitors of resistance-induced mediators and molecular signaling pathways.

In the case of cisplatin, the therapeutic efficiency of brain tumor treatment can be increased by using ezatiostat, an inhibitor of GSTP1. As mentioned previously, GSTP1 is involved in cisplatin resistance and metabolism in glioma cells [125]. Ezatiostat (g-glutamyl-S-(benzyl) cysteinyl-R-phenyl glycine diethyl ester) is a synthetic tripeptide analog prodrug of glutathione that selectively binds and inhibits GSTP1 [177]. Ezatiostat allows metabolites to selectively inhibit GSTP and phosphorylate c-Jun, which promotes apoptosis in malignant tumor cells [178,179]. Additionally, novel ezatiostat analogs interfere with the binding of GSTP1 to the three major MAP kinases (JNK, ERK, and p38) in various carcinomas. As a result, there is a difference in anticancer activity depending on the chemical structure of the analog of ezatiostat, and the difference is also based on the tumor cells [180,181]. In addition to ezatiostat, a study was conducted in 2020 on nipecotic acid ester, m-nitrophenyl-3-piperideinecarboxylate hydrochloride (MNPC) inhibitor, a dual inhibitor NAD(P)H quinone oxidoreductase 1, and GSTP1 for the treatment of GBM. It was observed that MNPC, a small molecular inhibitor of NQO1 and GSTP1, attacked the vulnerabilities generated by mutant EGFR, increasing the treatment efficacy of GBM [182,183]. Therefore, cisplatin chemotherapy using an inhibitor of GSTP1 can enhance the therapeutic efficiency in brain tumors.

In the case of carboplatin, the effectiveness of brain tumor treatment can be increased by using sorafenib, a drug that inhibits Mcl-1. Sorafenib is an EU-approved oral multikinase inhibitor used for the treatment of hepatocellular carcinoma [184,185]. Sorafenib inhibits cell surface tyrosine kinase receptor (vascular endothelial growth factor receptor) and intracellular serine/threonine kinase (Raf-1). These kinases are involved in tumor cell proliferation and tumor vessel formation [186]. In a previous study, sorafenib increased the treatment efficiency of acute promyelocytic leukemia (APL) by inhibiting Mcl-1. Sorafenib reversed p90RSK activation and GSK3β inactivation, blocked Mcl-1 increase and maintained suppressed Bcl-1 levels. As a result, it enhanced apoptosis in APL cells [187,188]. Sorafenib has also been shown to be effective in treating refractory differentiated thyroid cancer. It has been shown that sorafenib improved its anticancer effect by significantly increasing the effective drug concentration in thyroid cancer cell lines, TPC-1 and BCPAP [189]. Moreover, it was confirmed that the RAF/MEK/ERK signaling pathway was remarkably reduced by this inhibitor [190]. Sorafenib has also been reported to increase the therapeutic efficiency of anticancer response treatment in breast cancer and renal cell carcinoma [191,192,193].

Nathan B. Roberts identified STAT3 as a major regulator of oxaliplatin resistance. Studies have been conducted on STAT3 inhibitors in many carcinomas. STAT3 regulates the expression of genes that mediate cell survival, proliferation, and angiogenesis and is abnormally activated in many types of malignancies, including brain tumors [194,195]. In renal cell carcinoma, WP1066, a STAT3 inhibitor, increased treatment efficacy. WP1066 suppresses the expression of Bcl-2, HIF-1ɑ, and HIF-2ɑ, which induce hypoxia. It also inhibits the secretion of vascular endothelial growth factor and induces apoptosis [196,197,198]. In addition, a recent study in 2019 reported that the stemness of GBM was inhibited by the STAT3 inhibitor napabucasin (BB1608). Napabucasin inhibited the proliferation and invasion of GBM cell lines, U87MG and LN229. Napabucasin also blocked the NF-κB signaling pathway by downregulating RELA (p65), leading to cell cycle arrest and cell apoptosis [199,200]. It has also been reported that the STAT3 inhibitor, napabucasin, damages liver cancer stem cells and increases the therapeutic efficiency of chemotherapy in refractory colon cancer [201,202]. In the 40 years since the discovery of cisplatin, six drugs have been approved for the market and used to treat a variety of carcinomas, including brain tumors. Based on effective clinical results, further research is needed on the combination therapy of platinum-based anticancer drugs and various inhibitors to increase the efficiency of brain tumor treatment in the future.

**Figure 1:**
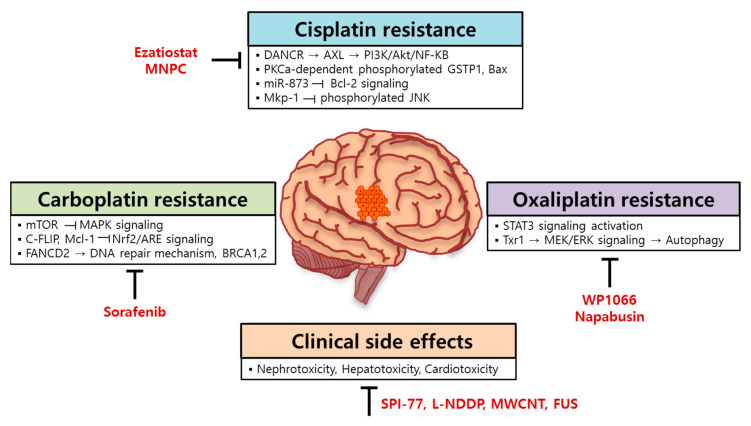
An overview of obstacles to overcome in order to increase the efficiency of platinum-based anticancer drugs against brain tumors. The figure summarizes the molecular mechanisms of resistance to platinum-based anticancer drugs in brain tumors. Ezatiostat, MNPC, sorafenib, WP1066, and napabucasin are drugs that can overcome resistance. It is possible to induce an anticancer response by improving the drug delivery system.

In addition to alleviating drug resistance, minimizing side effects of platinum-based drugs can increase their efficacy. These side effects can be avoided by efficient delivery systems achieved by cancer-specific targeting [203,204,205]. Efficient drug delivery systems include liposomes, dendrimers, polymers, and nanotubes. Liposomes (phospholipid bilayer vesicles) are one of the most promising and highly developed drug carriers for platinum drug delivery [206]. The liposome delivery method can improve the amount of drug in tumor cells by increasing vascular permeability and increasing the EPR effect [207]. Currently, liposome formulations developed for the efficient delivery of platinum-based drugs include SPI-77 and L-NDDP and showed excellent effects without toxicity in phase 1 clinical trials [208,209]. Studies have suggested that drug toxicity can be reduced by nano-formulation of platinum-based drugs [210]. Nanomedicine or nanotechnology-based chemotherapy has the potential for new cancer treatment approaches by improving drug delivery to tumors and reducing toxic side effects of drugs [211,212,213]. It has been reported that the therapeutic efficacy of oxaliplatin was improved by using hyaluronic acid–polymerized nanoparticles (DACHPt/HANP) [214,215]. Furthermore, MWCNT, a multi-walled carbon nanotube, was used to reduce side effects and maintain cisplatin’s ability to kill human lung cancer cells [216,217]. Recently, using the clinical device FUS, carboplatin has been delivered efficiently without contributing to the BBB. FUS is a promising, non-invasive method that can deliver drugs to the CNS by breaking down the BBB using microbubbles [218]. As a result of the study, it reduced the growth of gliomas without neurotoxicity and increased the survival rate [46]. Moreover, anticancer drug therapy has evolved from cytotoxic drugs to tumor-selective genomic and immune target drugs [219]. The complexity and heterogeneity of tumors has changed the cancer therapy paradigm [220]. This suggests that each patient needs personalized treatment and combination treatment [221]. Therefore, it is necessary to consider platinum-based drugs as a combination therapy to increase the treatment efficacy of brain tumors. We summarize an overview of obstacles to overcome for increasing the efficiency of platinum-based anticancer drugs against brain tumors (Figure 1).

On the other hand, efforts are being made to develop new platinum anticancer complexes to overcome cancer [222]. Platinum (IV) prodrug, which has improved the pharmacological properties of anticancer drugs, is more stable when administered orally, and has a lower tendency to react with proteins, thus reducing side effects [223,224]. One of the platinum (IV) prodrugs, satraplatin, is the first lipophilic platinum-based drug. It has been shown to overcome the defect in accumulation of cisplatin-resistant cell lines [224]. Because it is in clinical trials in combination with various drugs for the treatment of non-small cell lung cancer or advanced solid tumors, we hypothesize that it can be applied to brain tumor therapy [223].

## 6. Conclusions

In the field of chemotherapy, many efforts have been made to improve the treatment efficiency of brain tumors. However, the survival rate has not improved, and currently, there are not many chemotherapy options available to patients. This review summarizes the results and advantages of clinical treatment of brain tumors using platinum-based anticancer drugs. As another option for overcoming brain tumors, we focused on the limitations of the clinical application of these drugs and the signaling mechanisms that cause drug resistance. Studies have shown that the therapeutic efficacy of platinum-based anticancer drugs has more clinically significant results than TMZ, which is generally used to treat brain tumors. Furthermore, we also discussed the mechanism of drug resistance signaling by three drugs that have been applied to the treatment of brain tumors. We proposed the use of resistance-inducing factor inhibitor and major signaling pathway inhibitors as indirect methods to overcome platinum-based drug resistance. However, the molecular mechanisms of platinum-based drugs for the treatment of brain tumors remain unclear. The combined treatment of the resistance-inducing factor inhibitor and a platinum-based anticancer drug suggested in this review can be proposed as a method to optimize the treatment efficacy of brain tumors. However, reports on this combination are still lacking. Moreover, platinum-based drug efficacy can be maximized through the development of an efficient drug delivery system capable of penetrating the BBB. Therefore, it is necessary to revisit platinum-based drugs to improve the treatment efficacy of brain tumors.

## Figures and Tables

**Table 1:** The clinical trials of platinum-based anticancer drugs applied to brain tumor treatment.

Drug	Tumor Type	Number of Clinical Trial Participants	Major Findings	Reference
Cisplatin	Glioblastoma	30	On MRI, CT images, tumor growth was suppressed, and median survival was increased by 7–14 months.	[93,94,95,96]
	High-grade gliomas	10		
Carboplatin	Child brain Tumor	95	4 weeks, a good response was shown in 40 brain tumor patients. The median overall survival rate was 12 months.	[97,98,100,101]
	Gliomas	34		
	Recurrent glioblastomas	122		
Oxaliplatin	Refractory pediatric brain tumors	43	3 weeks, there was no CR, and PR (15 patients, 13.3%) was seen in the MRI images.	[102,104,105,106,109]
	Medulloblastoma, diffuse cranial glioma	11		

## Data Availability

No data were used in this review.
